# Location-Specific Hematoma Volume Cutoff and Clinical Outcomes in Intracerebral Hemorrhage

**DOI:** 10.1161/STROKEAHA.122.041246

**Published:** 2023-05-23

**Authors:** Kay-Cheong Teo, Sze-Man Fong, William C.Y. Leung, Ian Y.H. Leung, Yuen-Kwun Wong, Olivia M.Y. Choi, Ka-Keung Yam, Rachel C.N. Lo, Raymond T.F. Cheung, Shu-Leong Ho, Anderson C.O. Tsang, Gilberto K.K. Leung, Koon-Ho Chan, Kui-Kai Lau

**Affiliations:** Division of Neurology, Department of Medicine, Queen Mary Hospital (K.-C.T., S.-M.F., W.C.Y.L., I.Y.H.L., Y.-K.W., K.-K.Y., R.C.N.L., R.T.F.C., S.-L.H., K.-H.C., K.-K.L.), LKS Faculty of Medicine, The University of Hong Kong, Hong Kong SAR.; Research Center of Heart, Brain, Hormone and Healthy Aging (R.T.F.C., S.-L.H., K.-H.C., K.-K.L.), LKS Faculty of Medicine, The University of Hong Kong, Hong Kong SAR.; Division of Neurosurgery, Department of Surgery, Queen Mary Hospital (O.M.Y.C., A.C.O.T., G.K.K.L.), LKS Faculty of Medicine, The University of Hong Kong, Hong Kong SAR.; The State Key Laboratory of Brain and Cognitive Sciences, The University of Hong Kong, Hong Kong SAR (R.T.F.C., K.-H.C., K.-K.L.).

**Keywords:** cutoff, hematoma location, intracerebral hemorrhage, stroke outcome

## Abstract

Major intracerebral hemorrhage (ICH) trials have largely been unable to demonstrate therapeutic benefit in improving functional outcomes. This may be partly due to the heterogeneity of ICH outcomes based on their location, where a small strategic ICH could be debilitating, thus confounding therapeutic effects. We aimed to determine the ideal hematoma volume cutoff for different ICH locations in predicting ICH outcomes.

**Methods:**

We retrospectively analyzed consecutive ICH patients enrolled in the University of Hong Kong prospective stroke registry from January 2011 to December 2018. Patients with premorbid modified Rankin Scale score >2 or who underwent neurosurgical intervention were excluded. ICH volume cutoff, sensitivity, and specificity in predicting respective 6-month neurological outcomes (good [modified Rankin Scale score 0–2], poor [modified Rankin Scale score 4–6], and mortality) for specific ICH locations were determined using receiver operating characteristic curves. Separate multivariate logistic regression models were also conducted for each location-specific volume cutoff to determine whether these cutoffs were independently associated with respective outcomes.

**Results:**

Among 533 ICHs, the volume cutoff for good outcome according to ICH location was 40.5 mL for lobar, 32.5 mL for putamen/external capsule, 5.5 mL for internal capsule/globus pallidus, 6.5 mL for thalamus, 17 mL for cerebellum, and 3 mL for brainstem. ICH smaller than the cutoff for all supratentorial sites had higher odds of good outcomes (all *P*<0.05). Volumes exceeding 48 mL for lobar, 41 mL for putamen/external capsule, 6 mL for internal capsule/globus pallidus, 9.5 mL for thalamus, 22 mL for cerebellum, and 7.5 mL for brainstem were at greater risk of poor outcomes (all *P*<0.05). Mortality risks were significantly higher for volumes that exceeded 89.5 mL for lobar, 42 mL for putamen/external capsule, and 21 mL for internal capsule/globus pallidus (all *P*<0.001). All receiver operating characteristic models for location-specific cutoffs had good discriminant values (area under the curve >0.8), except in predicting good outcome for cerebellum.

**Conclusions:**

ICH outcomes differed with location-specific hematoma size. Location-specific volume cutoff should be considered in patient selection for ICH trials.

Intracerebral hemorrhage (ICH) is a deadly and debilitating form of stroke. Despite relentless efforts, effective treatment to improve functional outcomes following ICH has remained elusive.^1–7^ With the exception of the INTERACT-2 trial (The Second Intensive Blood Pressure Reduction in Acute Cerebral Hemorrhage Trial), where a significant positive shift in the modified Rankin Scale (mRS) was demonstrated with acute blood pressure (BP) lowering,^1^ acute therapeutic trials for ICH, which include BP lowering or hemostatic drugs administration to reduce hematoma expansion (HE) and surgical evacuation of hematoma, had failed to convincingly yield positive results.^1–7^ A possible explanation for these treatments’ lack of therapeutic benefit is the heterogeneity of ICH outcomes based on their location, where a small strategic ICH could be debilitating, thus confounding therapeutic effects.

**See related article, p 1558**

It is well recognized that ICH outcomes differ significantly depending on their location.^8–20^ Traditionally, ICH prognostication based on location is separated crudely into infratentorial and supratentorial.^21^ However, recent studies have demonstrated that the prognostication of ICH outcomes should be classified into more specific anatomic sites.^8–20^ Although studies have produced inconsistent results, for supratentorial ICH, the prognosis is generally better for lobar ICH^8–11^ but worse for thalamic.^12,13^ Importantly, since ICH volume is also another crucial modifier of ICH outcomes,^22^ it is becoming more evident that a location-specific ICH volume would better predict ICH outcomes.^19,20^ A small strategic bleed affecting the thalamus may have devastating neurological deficits, while a similar-sized ICH at the frontal lobe could have a more favorable neurological outcome.

Therefore, as the interaction between ICH location and volume significantly impacts neurological outcomes, using location-specific hematoma volumes for patient selection could, in theory, better guide the selection of patients who may best benefit from treatment. Herein, we aimed to determine the ideal location-specific volume cutoffs in predicting ICH outcomes at different ICH sites.

## Methods

### Data Availability Statement

Anonymized data pertaining to the research presented will be made available from the corresponding author upon reasonable request.

### Study Design and Participants

We retrospectively analyzed consecutive ICH patients enrolled in the University of Hong Kong prospective stroke registry from January 2011 to December 2018. We included all primary ICH patients aged ≥18 who presented to Queen Mary Hospital, Hong Kong, within 48 hours of symptom onset or last seen well (LSW) time. ICH diagnosis was confirmed by computed tomography (CT) scan of the brain. Individuals who underwent neurosurgical intervention, with premorbid mRS score of >2, secondary ICH, multiple ICH locations, or had a recurrent ICH/stroke within 6 months of index ICH were excluded. The study protocol was approved by the institutional review boards of our institution. Written informed consent was obtained from all patients, or their next of kin for patients who could not consent during enrollment into the stroke registry. The study was reported in accordance with the STROBE (Strengthening the Reporting of Observational Studies in Epidemiology) reporting guidelines.

### Management of ICH

All patients were managed according to our hospital’s established stroke pathway, which is regularly updated in line with the American Heart Association ICH guidelines.^23,24^ In brief, the stroke pathway is activated, and our stroke team is notified by the Accident and Emergency Department for all patients with suspected stroke. An urgent CT brain is performed, and neurosurgery will be consulted in cases of ICH. The decision to proceed with surgery is at the discretion of the neurosurgeon on-call after discussion with the patient or next of kin. Indications for surgical treatment include ICH within 1 cm of the surface with obtunded Glasgow Coma Scale or significant mass effect, hydrocephalus, and cerebellar ICH with brainstem compression or hydrocephalus. Patients deemed unsuitable for surgery are transferred to our high-dependency stroke unit under the care of a multidisciplinary team. A standardized management protocol with a BP reduction target of systolic BP 140 mm Hg is adopted. Upon stabilization, patients who require further rehabilitation are transferred to a designated stroke rehabilitation unit at Tung Wah Hospital, Hong Kong.

### Variables and Outcomes

Demographic data, social, and medical histories were collected by trained study staff through in-person interviews of patients (and reliable informants) and a review of electronic medical records at the time of enrollment. Clinical data captured included Glasgow Coma Scale and BP on admission.

Six-month mRS scores were determined based on electronic records of clinic or rehabilitation visits at 6 months (±1 month) after index ICH. We defined good outcome as mRS score 0 to 2 and poor outcome as mRS score 4 to 6.

### Imaging Analysis

All CT brain images were reviewed blinded to clinical outcomes. CT scans were analyzed to determine ICH location, hematoma volume, and the presence of intraventricular hemorrhage (IVH). ICH location was classified as lobar, external capsule (EC), putamen, globus pallidus (GP), internal capsule (IC), thalamus, caudate head, cerebellum, or brainstem. For large ICH with overlapping sites, the CT images were reviewed by 2 experienced stroke neurologists (K.C.T. and K.K.L.) to determine the exact ICH location. However, as it is often difficult to differentiate EC from putamen, and GP from IC due to the close proximity of these structures, we grouped EC and putamen, and GP and IC together. Hematoma volume was determined using the ABC/2 formula.^25^ If multiple CTs were performed within 48 hours of admission, the largest ICH volume was recorded for analysis. The severity of IVH was scored based on the Graeb score.^26^ The Graeb score is a semiquantitative measure of IVH, ranging from 0 to 12 points, with the higher scores indicating increased IVH volumes.

### Statistical Methods

Statistical analyses were performed using SPSS version 28.0 and Prism version 6.0. Descriptive statistics are presented either as mean and SD, or median with interquartile range for continuous variables, and number and percentage of the subtotal for categorical variables. Categorical variables were compared using χ^2^ or Fisher exact tests, and continuous variables using the Kruskal-Wallis or Student *t* test. Patients with caudate head ICH were excluded due to their small number (n=17).

Multivariate logistic regression analysis was performed to determine the association of ICH location with poor outcome and mortality. Each individual ICH location (lobar, thalamus, IC/GP, putamen/EC, brainstem, and cerebellum) was entered into separate multivariate logistic regression models to determine the association between different locations and outcomes. The multivariate regression models included age, ICH volume (log-transformed), and covariates with *P*<0.2 in univariable analyses with backward elimination to arrive at a minimal model that included only variables associated at *P*<0.1. Glasgow Coma Scale was classed as ≥9 or <9 based on the FUNC score,^18^ while the Graeb score was categorized as ≥5 or <5 since the former was associated with poor outcomes in ICH.^27^ An interaction term was next added to the regression model to test whether there was a significant interaction between ICH location and volume. As outcomes differ between lobar, deep, and infratentorial ICH, additional multivariate logistic regression models were performed specifically for deep and infratentorial ICH.

The optimal ICH volume cutoff (to the closest 0.5 mL), sensitivity, and specificity in predicting the different outcomes of interest (good outcome [mRS score 0–2], poor outcome [mRS score 4–6], and mortality at 6 months) for different ICH locations were determined using receiver operating characteristic (ROC) curves. Separate multivariate logistic regression models were conducted for each location-specific hematoma volume cutoff with the respective outcome of interest (good outcome versus mRS score 3–6; poor outcome versus mRS score 0–3; mortality versus mRS score 0–5). We included age and covariates with *P*<0.2 from the univariable analyses into the multivariate regression model with backward elimination to arrive at a final model including only the location-specific cutoff and variables associated at *P*<0.1. All significance tests were 2-tailed, and significance was set at *P*<0.05. Data were reported with odds ratio (OR) and 95% CI.

### Sensitivity Analyses

Separate ROC analyses were performed for symptoms onset/LSW to CT time ≤6 and >6 hours for each ICH location to determine whether the cutoffs varied according to time windows. The ROC curves based on the time windows for each location were compared using the DeLong test to evaluate whether they differed significantly.

We also performed sensitivity analyses to assess how the sensitivity and specificity of each location-specific volume cutoff based on the primary analysis varied after including patients who underwent surgery, and to determine whether these cutoffs remained independently associated with outcomes after including these patients. The sensitivity and specificity of all location-specific cutoffs were calculated using the ROC curve, and multivariate logistic regression analyses were performed on each location-specific volume cutoff. Similarly, for respective location and outcome of interest, covariates with *P*<0.2 from the univariable analyses were entered into a multivariate regression model with backward elimination to arrive at a final model including only the location-specific cutoff and variables associated at *P*<0.1.

## Results

**Figure 1.**
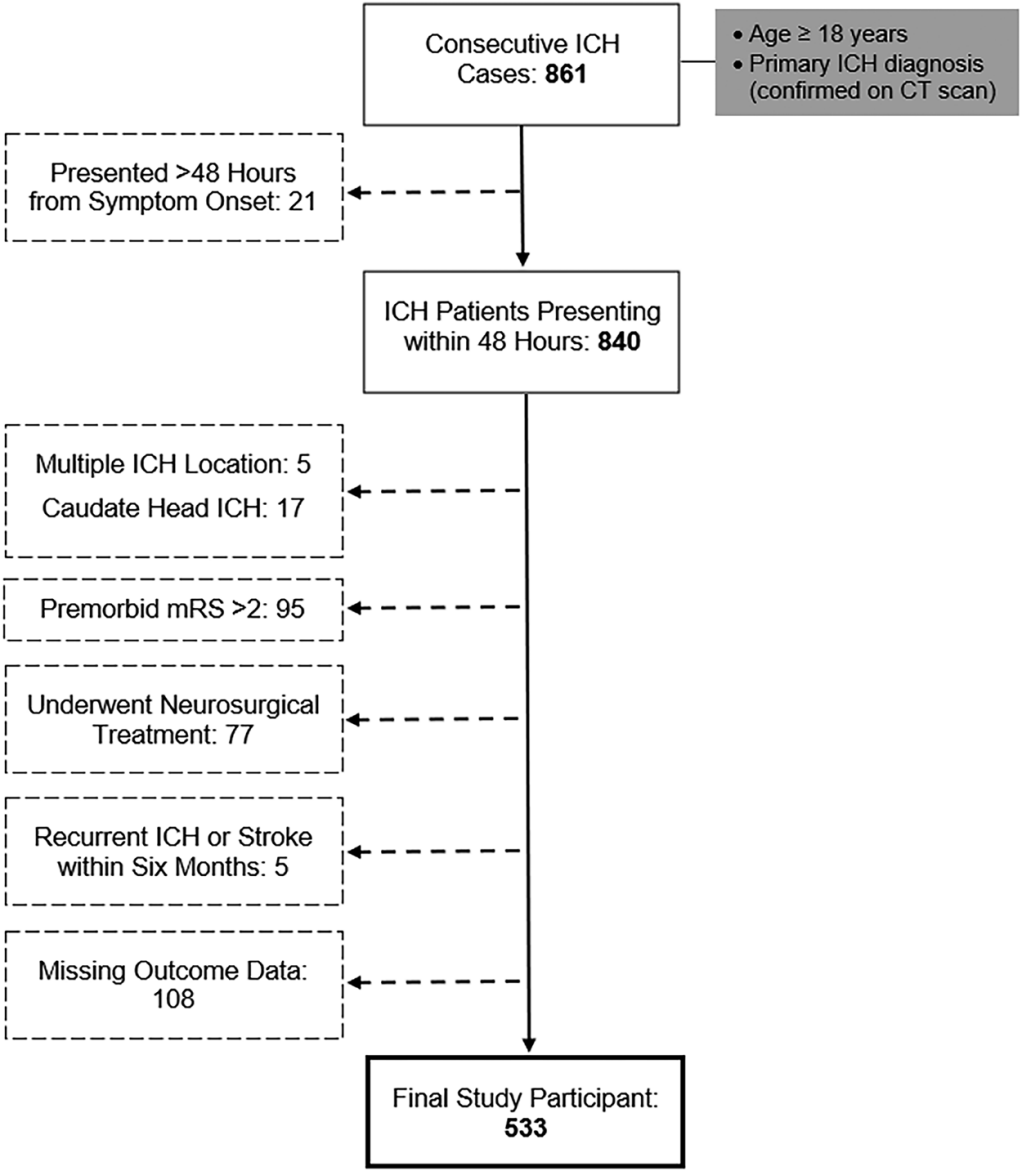
**Flow diagram of study inclusion and exclusion criteria.** CT indicates computed tomography; ICH, intracerebral hemorrhage; and mRS, modified Rankin Scale.

Of 861 consecutive ICH patients included in the stroke registry during the study period, 533 were eligible to the current analysis (Figure 1). As expected, compared with patients with mRS score ≥3 at 6 months, those with good outcomes were younger, had higher admission Glasgow Coma Scale score, smaller ICH volume and IVH score, longer symptom onset/LSW to CT time and were less likely to have infratentorial ICH. They were also more likely to present with an ICH located at the putamen/EC (Table 1). There were no significant differences in the clinical or outcome measures between patients with ICHs presumably located at the IC and GP (Table S1) or between putamen and EC (Table S2).

**Table 1.**
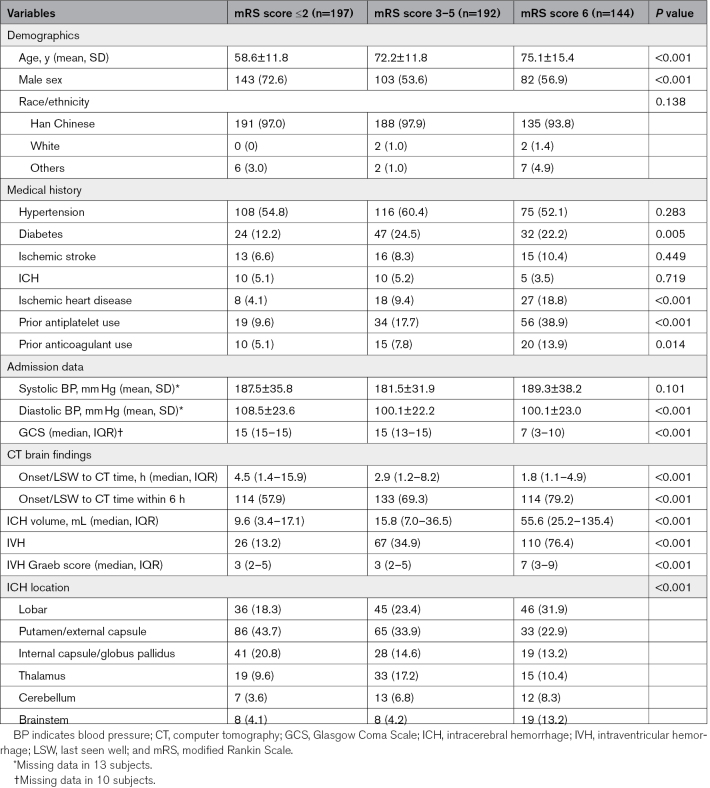
Study Participants’ Characteristics According to the 6-Month Modified Rankin Scale

### Clinical Characteristics and Outcomes Based on ICH Location

The clinical characteristics and outcomes differed significantly according to ICH location (Table 2). Notably, patients with lobar ICH were older, had larger ICH volumes, and were less likely to have an IVH. Patients with IC/GP ICHs and putamen/EC ICHs also significantly differ, where IC/GP ICHs had smaller hematoma volumes and were more likely to have IVH. Poor outcome and mortality at 6 months were the highest for infratentorial-located ICHs.

**Table 2.**
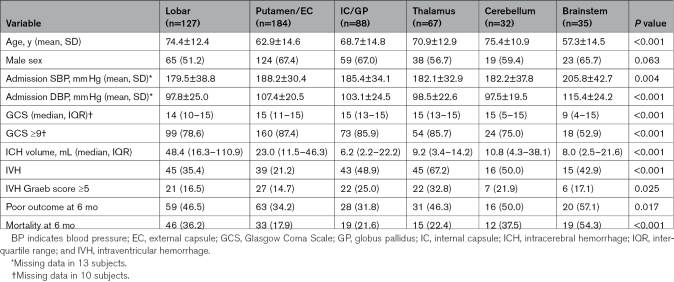
Clinical Characteristics and Outcomes Based on ICH Location

### Analyses of ICH Location and 6-Month Outcomes

Lobar ICH was associated with a significantly lower risk of poor outcome (adjusted odds ratio [aOR], 0.13 [95% CI, 0.06–0.29]). The risk of poor outcome was highest for thalamic (aOR, 2.66 [95% CI, 1.17–6.08]) and brainstem ICH (aOR, 8.87 [95% CI, 2.38–33.06]). In a subgroup analysis of only deep ICH, the risk of poor outcome remained the greatest for thalamic ICH but was reduced for putamen/EC ICH (aOR, 0.18 [95% CI, 0.06–0.48]; Table 3).

**Table 3.**
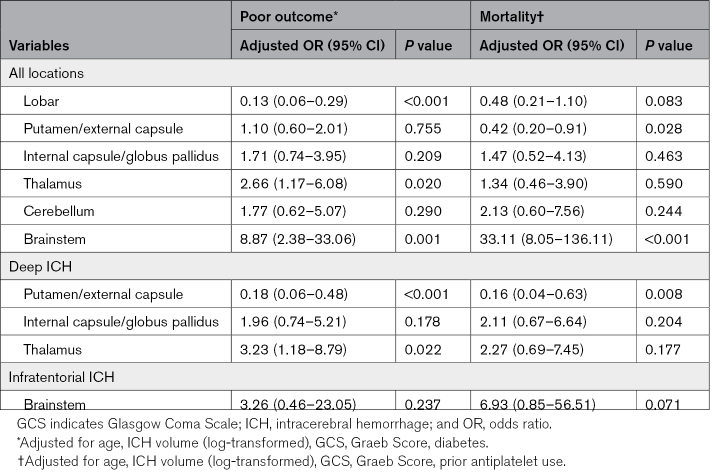
Multivariate Analyses of ICH Locations and 6-Month Outcomes

For mortality, patients with putamen/EC ICH had a lower risk (aOR, 0.42 [95% CI, 0.20–0.91]), while the risk was increased for brainstem ICH (aOR, 33.11 [95% CI, 8.05–136.11]; Table 3).

Interaction analysis demonstrated a significant interaction between ICH volume and location for both poor outcome and mortality in adjusted models (*P*_interaction_<0.05).

There was otherwise no association of laterality with outcome in univariate analysis (all *P*>0.80), so this was not entered into the multivariate regression model.

### Location-Specific Hematoma Volume Cutoff and the Association With Clinical Outcomes

The respective location-specific volume cutoffs, sensitivity, and specificity in predicting different clinical outcomes are presented in Table 4. All area under the curves of the location-specific cutoff for good outcome, poor outcome, and mortality were >0.8, except for good outcome for cerebellum (area under the curve, 0.623).

**Table 4.**
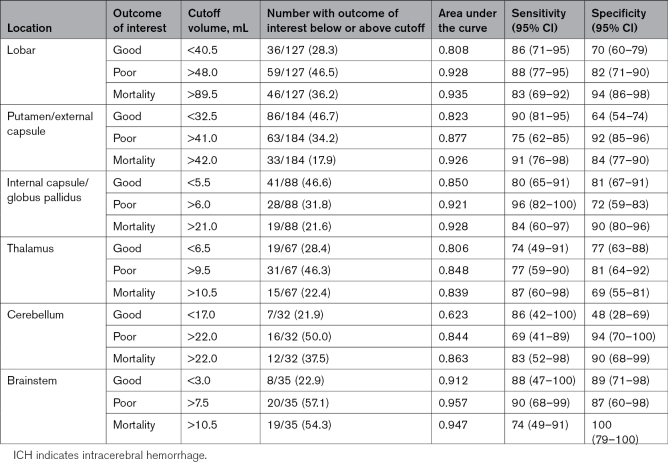
ICH Volume Cutoffs for Specific Outcome of Interest Based on ICH Location

**Figure 2.**
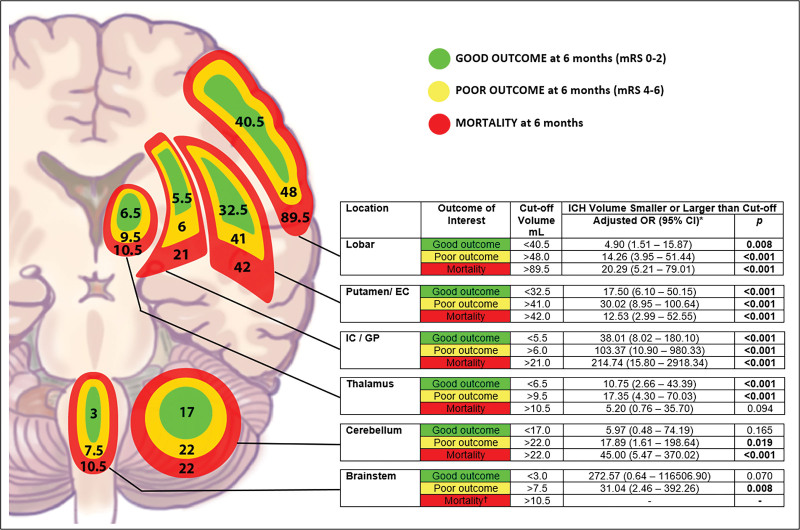
**Location-specific hematoma volume cutoff and neurological outcome.** The location-specific volume cutoff for predicting neurological outcomes of different intracerebral hemorrhage (ICH) locations are shown. Green represents good outcome (modified Rankin Scale [mRS] score 0–2), yellow represents poor outcome (mRS score 4–6), and mortality is colored red. The odds for good outcomes were significantly higher when ICH volumes were smaller than the location-specific cutoffs for all supratentorial ICH sites (all *P*<0.05). For poor outcome, the risk for all ICH locations was greater when volumes exceeded the respective cutoffs (all *P*<0.05). Mortality risks were also higher for volumes larger than cutoffs for lobar, putamen/external capsule (EC), internal capsule (IC)/globus pallidus (GP), and cerebellum (all *P*<0.001). *Multivariate regression analysis of respective outcome and location is available in Table S3. †Regression analysis cannot be performed as all brainstem ICH patients with volume >10.5 mL died at 6 mo.

The odds for good outcomes were significantly higher for ICH with volume smaller than the location-specific cutoff for all supratentorial ICH locations (all *P*<0.05; Figure 2). For poor outcome, the risk for all ICH locations were greater when ICH volumes exceeded the cutoffs (all *P*<0.05; Figure 2). Mortality risks were significantly higher for volumes that exceeded 89.5 mL for lobar, 42 mL for putamen/EC, 21 mL for IC/GP, and 22 mL for cerebellum (all *P*<0.001; Figure 2).

### Sensitivity Analyses for Location-Specific Hematoma Volume Cutoff

Table S4 shows the respective location-specific volume cutoffs, sensitivity, and specificity in predicting clinical outcome, stratified by symptom onset/LSW to CT time of ≤6 versus >6 hours. There was no significant difference between the ROC curves of both time windows for all locations (all *P*>0.05 for DeLong test). Patients presenting >6 hours after symptom onset/LSW tended to have smaller ICH (median volume 10.7 versus 21.8 mL; *P*<0.001)

After including the 77 patients who had undergone surgery, the sensitivity and specificity for respective location-specific cutoffs are presented in Table S5. These cutoffs remained significantly associated with outcomes of interest when compared to the primary analysis (Table S6).

## Discussion

Our study has demonstrated the critical interaction between ICH location and volume with neurological outcomes. The varying location-specific volume cutoffs in predicting neurological outcomes for different ICH sites reflect the heterogeneity of ICH outcomes based on location. The prognosis of a small ICH at the thalamus or IC/GP is much worse than that for a similar-sized ICH at the putamen/EC or lobar region. These findings may have important implications in patient selection for future ICH trials.

One of the important caveats of previous therapeutic trials for acute ICH treatment is that most have not accounted for the heterogeneity of outcomes based on ICH location.^1–5^ The interaction between ICH location and volume with outcome could be more crucial in acute therapeutic trials for ICH than those for ischemic stroke, as the therapeutic benefit of acute treatment of ICH is not as profound as that of reperfusion therapies for ischemic stroke. Successful reperfusion therapy for ischemic stroke may completely reverse brain injury. On the contrary, acute treatment of ICH, either by reducing HE or surgical evacuation, will not reverse brain injury but only prevent further damage. Hence, the interaction between ICH location and volume with neurological outcomes will drastically confound treatment effects. For HE reduction trials, most trials including ATACH-2 (Antihypertensive Treatment of Acute Cerebral Hemorrhage II), TICH-2 (Tranexamic Acid for Hyperacute Primary Intracerebral Haemorrhage), and SPOTLIGHT/STOP-IT (“Spot Sign” Selection of Intracerebral Hemorrhage to Guide Hemostatic Therapy/The Spot Sign for Predicting and Treating ICH Growth Study) were successful in reducing hematoma expansion but failed to demonstrate outcome benefit.^2–5^ Our study may provide some valuable insight into these findings. Based on our results, a patient with thalamic ICH >9.5 mL volume would be highly unlikely to achieve a good neurological outcome, even with the best trial drug that could stop HE immediately. In comparison, a good outcome would be expected for a 20 mL putaminal ICH regardless of any treatment effect. Hence, location-specific volume cutoffs should be applied based on the primary outcome of interest for patient selection in clinical trials. For example, in an HE reduction trial that aims to reduce the rate of poor outcome, patients with ICH volumes smaller than the location-specific volume cutoffs for poor outcome will be ideal study candidates (48.0 mL for lobar, 41 mL for putamen/EC, 6 mL for IC/GP, and 9.5 mL for thalamus), in which prevention of HE beyond these cutoffs would likely translate to clinical benefit. ICH located at the putamen/EC or lobar region would be the ideal site for these trials as there is a larger volume difference between good, poor outcomes and mortality, allowing more flexibility for patient selection and reducing the confounding effect of unaccounted hematoma expansion between the time of brain imaging and study drug administration on neurological outcome. These areas are also less likely to have IVH and brain stem injury,^28^ which also would affect neurological outcomes of ICH.

Similarly, location-specific volume cutoff can be applied in patient selection for surgical evacuation. Surgical evacuation theoretically improves neurological outcomes by reducing secondary brain injury due to the inflammatory response driven by the hematoma, mass effect, and elevated intracranial pressure.^29,30^ The STICH-II trial (Surgical Trial in Lobar Intracerebral Haemorrhage) included only lobar ICHs,^6^ while deep ICHs (mainly putamen/EC) were also included in the MISTIE III trial (Minimally Invasive Surgery With Thrombolysis in Intracerebral Hemorrhage Evacuation Phase III).^7^ The median ICH volume was around 40 mL for both of these studies. Around 40% to 50% of study subjects in the nonsurgical arm had favorable neurological outcomes (mRS score 0–3).^6,7^ These findings were compatible with our results, where the odds of poor outcome (mRS score 4–6 in the current study) were lower for lobar ICH with hematoma volume ≤48 mL, and ≤41 mL for putamen/EC ICH. Since increasing hematoma size augments secondary brain injury in ICH,^29,30^ we postulate that there may be a critical hematoma volume associated with maximal secondary brain injury and consequent poor outcome. Applying a volume cutoff >48 mL for lobar and >41 mL for putamen/EC for patient selection for hematoma evacuation may capture patients who will best benefit from surgery. In the exploratory secondary results of MISTIE III, a favorable outcome in clot size reduction to 15 mL or less in the MISTIE group was predominantly seen in patients with initial ICH >45 mL.^7^

Our findings are consistent with previously published literature on location-specific ICH outcomes.^8–13^ In a recently published article from the ERICH and ATACH-2 cohort on deep ICH, hematoma volume cutoff for poor outcome for thalamic ICH was 8 mL (9.5 mL in our study) and 18 mL for basal ganglia ICH.^19^ We provided 2 separate cutoff volumes for basal ganglia ICH, IC/GP, and putamen/EC, respectively, as the latter tend to have larger ICH volume (median volume 23.0 versus 6.2 mL; *P*<0.001). Another important finding is that lobar ICH was associated with favorable outcomes despite its larger volume. Since motor impairment is one of the most crucial factors for stroke recovery,^31^ a plausible explanation for the favorable outcome noted in lobar ICH is that the predilection for motor pathway involvement is less compared with other ICH locations due to its large area. We postulate that the location-specific volume cutoff would differ further for different ICH sites in the lobar region (frontal, parietal, temporal, occipital), as there is a rostrocaudal gradient in functional outcome for lobar ICH, in which occipital ICH has the best outcome.^32^ Nonetheless, we could not perform such an analysis due to the limited numbers.

Our study has several limitations. First, this was a retrospective analysis of a prospective stroke cohort. However, as we recruited consecutive ICH patients treated in a single center, selection and outcome bias were limited. Second, our cohort size is limited, especially for infratentorial and caudate ICH. Our preliminary analysis demonstrated that caudate ICH is a distinct ICH site with definite IVH, which was usually severe and with high mortality, but the small sample size (n=17) limited further analysis of this site. For infratentorial ICH, the small numbers led to a wide 95% CI for ROC and regression analyses. Third, we employed the ABC/2 formula to calculate ICH volume rather than planimetric analysis. However, the formula is a well-established method to calculate ICH volume and is more practical for patient selection for trials.^33^ Forth, the symptom onset/LSW to CT time may affect the location-specific cutoff values, as early-presenting patients tend to have hematoma expansion, and late-arriving patients tend to have smaller ICH. Inherently, the cutoff values for late-arriving patients tended to be smaller. However, the potential confounding effect of the time window on the relationship between location-specific hematoma volume and outcome was likely minimized as we utilized the largest ICH volume recorded for all patients (for those with hematoma expansion, the largest ICH volume was used), rather than the volume from the first brain CT. Finally, our results are derived from a single center, where the predominant ethnicity was Han Chinese. Further studies are required to verify the generalizability of our results to other populations.

Our study displays numerous strengths. First, we present a novel idea for patient selection based on location-specific ICH volume cutoff due to the significant heterogeneity of ICH outcomes based on location. This may be why ICH trials have repeatedly failed to demonstrate therapeutic benefit in improving functional outcomes. Importantly, all the ROC models for location-specific cutoffs had good discriminant values, especially for supratentorial ICH, where these cutoffs were independently associated with different outcomes of interest. Finally, our patients were treated under a standardized stroke pathway from a single center, reducing the treatment and outcome bias.

In summary, we demonstrate significant heterogeneity in ICH outcomes based on location-specific hematoma volume and present location-specific volume cutoffs for different neurological outcomes of interest. Patient selection based on location-specific volume cutoff should be considered in future ICH trials.

## Article Information

### Acknowledgments

Drs Teo, Chan, and Lau performed concept and design. All authors performed acquisition, analysis, or interpretation of data. Drs Teo and Fong performed drafting of the article. All authors performed critical revision of the article for important intellectual content. Drs Teo and Wong performed statistical analysis. Drs Teo, Chan, A.C.O. Tsang, and Lau obtained funding. Choi and Dr Wong provided administrative, technical, or material support. Dr Teo had full access to all of the data in the study and take responsibility for the integrity of the data and the accuracy of the data analysis.

### Sources of Funding

The authors’ work on this study was supported by funding from the Research Fund Secretariat of the Food and Health Bureau, The Government of the Hong Kong SAR. The funding entities had no role in the design or conduct of the study; collection, management, analysis, or interpretation of the data; preparation, review, or approval of the article; and decision to submit the article for publication.

### Disclosures

Dr Teo is supported by the Hong Kong Neurological Society Scholarship for Young Neurologist. Dr Lau is supported by the Innovation and Technology Bureau, Research Grants Council, The Government of the Hong Kong SAR, Amgen, Boehringer Ingelheim, Eisai and Pfizer; and has consulted for Amgen, Boehringer Ingelheim, Daiichi Sankyo and Sanofi; all of whom are unrelated to the current work.

### Supplemental Material

STROBE checklist

Tables S1–S6

## Supplementary Material


