# Supplementary material for: Location-Specific Hematoma Volume Cutoff and Clinical Outcomes in Intracerebral Hemorrhage
Source: Stroke. 2023 May 23;54(6):1548–57. doi: 10.1161/STROKEAHA.122.041246 (PMC10266339; doi:10.1161/STROKEAHA.122.041246)
Supplement: Supplementary file 1 [file str-54-1548-s001.pdf]

Table S1: Clinical Characteristics and Outcomes of Internal Capsule and Globus Pallidus ICH

Variables	Internal Capsule (n = 70)	Globus Pallidus (n = 18)	p
Age, year (mean, SD)	69.6 ± 14.5	65.3 ± 15.6	0.272
Male Sex	47 (67.1)	12 (66.7)	1.000
History of Hypertension	48 (68.6)	13 (72.2)	1.000
History of Diabetes Mellitus	11 (15.7)	2 (11.1)	1.000
History of Ischemic Stroke	9 (12.9)	2 (11.1)	1.000
History of ICH	5 (7.1)	3 (16.7)	0.352
History of Ischemic Heart Disease	3 (4.3)	1 (5.6)	1.000
Prior antiplatelet use	17 (24.3)	3 (16.7)	0.753
Prior anticoagulant use	3 (4.3)	2 (11.1)	0.270
Admission Systolic BP, mmHg (mean, SD)	183.8 ± 34.2	191.8 ± 33.7	0.377
Admission Diastolic BP, mmHg (mean, SD)	102.7 ± 25.2	104.8 ± 22.2	0.750
Admission GCS (median, IQR)	15 (13 – 15)	15 (12 – 15)	0.367
ICH volume, mL (median, IQR)	6.2 (2.1 – 22.0)	6.5 (2.2 – 24.9)	0.729
Intraventricular Extension	39 (55.7)	4 (22.2)	0.016
IVH Graeb score (median, IQR)	5 (3 – 8)	3 (1 – 9)	0.292
Poor Outcome at six months	22 (31.4)	6 (33.3)	1.000
Mortality at six months	15 (21.4)	4 (22.2)	1.000

Abbreviations: BP = Blood Pressure, GCS = Glasgow Coma Scale, ICH = Intracerebral Hemorrhage, IVH = Intraventricular Hemorrhage

Table S2: Clinical Characteristics and Outcomes of Putamen and External Capsule ICH

Variables	Putamen (n = 123)	External Capsule (n = 61)	p
Age, year (mean, SD)	63.1 ± 14.0	62.4 ± 15.9	0.781
Male Sex	83 (67.5)	41 (67.2)	0.971
History of Hypertension	60 (48.8)	30 (49.2)	0.959
History of Diabetes Mellitus	15 (12.2)	14 (23.0)	0.059
History of Ischemic Stroke	8 (6.5)	2 (3.3)	0.500
History of ICH	2 (1.6)	2 (3.3)	0.601
History of Ischemic Heart Disease	12 (9.8)	7 (11.5)	0.798
Prior antiplatelet use	18 (14.6)	8 (13.1)	1.000
Prior anticoagulant use	13 (10.6)	2 (3.3)	0.150
Admission Systolic BP, mmHg (mean, SD)	185.6 ± 30.0	193.4 ± 31.0	0.104
Admission Diastolic BP, mmHg (mean, SD)	106.3 ± 19.9	109.5 ± 21.6	0.320
Admission GCS (median, IQR)	15 (11 – 15)	15 (10 – 15)	0.616
ICH volume, mL (median, IQR)	25.0 (12.2 – 46.8)	20.3 (11.0 – 42.8)	0.569
Intraventricular Extension	28 (22.8)	11 (18.0)	0.460
IVH Graeb score (median, IQR)	6 (3 – 8)	7 (3 – 8)	0.843
Poor Outcome	46 (37.4)	17 (27.9)	0.200
Mortality	24 (19.5)	9 (14.8)	0.541

Abbreviations: BP = Blood Pressure, GCS = Glasgow Coma Scale, ICH = Intracerebral Hemorrhage, IVH = Intraventricular Hemorrhage

Table S3. Respective Multivariate Analysis of Factors Associated with Outcome of Interest based on ICH Location

Location	Outcome of Interest	Variables	Adjusted Odds Ratio (95% Confidence Interval)	*p*
Lobar	Good outcome	ICH volume < 40.5 ml	4.90 (1.51 – 15.87)	0.008
		Every 10 years increase in age	0.42 (0.26 – 0.67)	<0.001
		GCS score ≥ 9	8.75 (0.87 – 87.94)	0.065
	Poor outcome	ICH volume > 48.0 ml	14.26 (3.95 – 51.44)	<0.001
		Every 10 years increase in age	5.21 (2.16 – 12.60)	<0.001
		GCS score ≥ 9	0.003 (0.00 – 0.09)	<0.001
	Mortality	ICH volume > 89.5 ml	20.29 (5.21 – 79.01)	<0.001
		Every 10 years increase in age	2.40 (1.17 – 4.91)	0.017
		GCS score ≥ 9	0.01 (0.00 – 0.19)	0.002
Putamen/ External Capsule	Good outcome	ICH volume < 32.5 ml	17.50 (6.10 – 50.15)	<0.001
		Every 10 years increase in age	0.28 (0.19 – 0.44)	<0.001
		IVH Graeb score ≥ 5	0.13 (0.02 – 0.93)	0.042
	Poor outcome	ICH volume > 41.0 ml	30.02 (8.95 – 100.64)	<0.001
		Every 10 years increase in age	2.86 (1.86 – 4.39)	<0.001
		IVH Graeb score ≥ 5	45.73 (6.11 – 342.29)	<0.001
		Known ischemic heart disease	4.33 (0.96 – 19.63)	0.057
	Mortality	ICH volume > 42.0 ml	12.53 (2.99 – 52.55)	<0.001
		Every 10 years increase in age	1.72 (1.05 – 2.79)	0.030
		IVH Graeb score ≥ 5	15.06 (2.46 – 92.33)	0.003
		GCS score ≥ 9	0.14 (0.02 – 0.99)	0.049
Internal Capsule/ Globus Pallidus	Good outcome	ICH volume < 5.5 ml	38.01 (8.02 – 180.10)	<0.001
		Every 10 years increase in age	0.36 (0.20 – 0.65)	<0.001
	Poor outcome	ICH volume > 6.0 ml	103.37 (10.90 – 980.33)	<0.001
		Every 10 years increase in age	3.12 (1.70 – 5.73)	<0.001
	Mortality	ICH volume > 21.0 ml	214.74 (15.80 – 2918.34)	<0.001
		Every 10 years increase in age	5.33 (1.75 – 16.24)	0.003
Thalamus	Good outcome	ICH volume < 6.5 ml	10.75 (2.66 – 43.39)	<0.001
		Every 10 years increase in age	0.43 (0.23 – 0.78)	0.005
	Poor outcome	ICH volume > 9.5 ml	17.35 (4.30 – 70.03)	<0.001
		Every 10 years increase in age	2.06 (1.22 – 3.48)	0.007
	Mortality	ICH volume > 10.5 ml	5.20 (0.76 – 35.70)	0.094
		Every 10 years increase in age	2.54 (1.08 – 5.93)	0.032
		GCS score ≥ 9	0.005 (0.00 – 0.15)	0.002
		Male Sex	11.29 (0.90 – 142.23)	0.061
Cerebellum	Good outcome	ICH volume < 17.0 ml	5.97 (0.48 – 74.19)	0.165
		Every 10 years increase in age	0.42 (0.17 – 1.01)	0.053
	Poor outcome	ICH volume > 22.0 ml	17.89 (1.61 – 198.64)	0.019
		Prior use of antiplatelet drug	7.10 (0.87 – 57.62)	0.067
	Mortality	ICH volume > 22.0 ml	45.00 (5.47 – 370.02)	<0.001
Brainstem	Good outcome	ICH volume < 3.0 ml	272.57 (0.64 – 116506.90)	0.070
		Every 10 years increase in age	0.19 (0.03 – 1.19)	0.077
	Poor outcome	ICH volume > 7.5 ml	31.04 (2.46 – 392.26)	0.008

Abbreviations: GCS=Glasgow Coma Scale, ICH=Intracerebral Hemorrhage, IVH=Intraventricular Hemorrhage

Table S4: Location-specific Hematoma Volume Cut-offs for Specific Outcome of Interest when Stratified based on Symptoms/ LSW to CT Time

Location	Outcome of Interest	Symptoms/ LSW to CT Time within Six Hours					Symptoms/ LSW to CT Time > Six Hours				
		Cut-off volume, mL	Number with Outcome of Interest Below/Above Cut-off	AUC	Sensitivity (95% CI)	Specificity (95% CI)	Cut-off volume, mL	Number with Outcome of Interest Below/Above Cut-off	AUC	Sensitivity (95% CI)	Specificity (95% CI)
Lobar	Good outcome	<28.0	14/77 (18.2)	0.829	79 (49-95)	81 (69-90)	<40.5	22/50 (44.0)	0.755	91 (71-98)	61 (41-79)
	Poor outcome	>48.0	47/77 (61.0)	0.939	91 (80-98)	83 (65-94)	>42.5	12/50 (24.0)	0.886	83 (52-98)	79 (32-90)
	Mortality	>89.5	40/77 (51.9)	0.922	83 (67-93)	92 (78-98)	>108.0	6/50 (12.0)	0.955	83 (36-100)	100 (92-100)
Putamen/ External Capsule	Good outcome	<32.5	62/144 (43.1)	0.848	89 (78-95)	70 (58-79)	<28.5	24/40 (60.0)	0.680	88 (68-97)	50 (25-75)
	Poor outcome	>41.0	50/144 (34.7)	0.924	82 (69-91)	91 (85-96)	>28.5	13/40 (32.5)	0.724	62 (32-86)	89 (71-98)
	Mortality	>42.0	28/144 (19.4)	0.931	93 (77-99)	82 (74-88)	>53.0	5/40 (12.5)	0.903	80 (28-99)	97 (85-100)
Internal Capsule/ Globus Pallidus	Good outcome	<5.5	22/60 (36.7)	0.830	72 (50-89)	82 (66-92)	<6.0	19/28 (67.9)	0.883	89 (67-99)	78 (40-97)
	Poor outcome	>17.0	22/60 (36.7)	0.920	77 (55-92)	95 (82-99)	>6.0	6/28 (21.4)	0.939	100 (54-100)	86 (65-97)
	Mortality	>21.0	14/60 (23.3)	0.941	93 (66-100)	89 (76-97)	>6.0	5/28 (17.9)	0.930	100 (48-100)	83 (61-95)
Thalamus	Good outcome	<12.0	10/40 (25.0)	0.827	100 (69-100)	53 (34-72)	<6.5	9/27 (33.3)	0.765	89 (52-100)	72 (47-90)
	Poor outcome	>18.0	21/40 (52.5)	0.837	52 (30-74)	100 (82-100)	>10.0	10/27 (37.0)	0.859	80 (44-97)	94 (71-100)
	Mortality	>18.0	10/40 (25.0)	0.857	80 (44-97)	90 (73-98)	>10.5	5/27 (18.5)	0.827	80 (28-99)	82 (60-95)
Cerebellum	Good outcome	<30.0	4/18 (22.2)	0.768	100 (40-100)	64 (35-87)	<7.0	3/14 (21.4)	0.515	100 (29-100)	45 (17-77)

	Poor outcome	>30.0	11/18 (61.1)	0.948	81 (48-98)	100 (59-100)	>10.0	5/14 (35.7)	0.644	60 (15-95)	89 (52-100)
	Mortality	>30.0	9/18 (50.0)	0.901	89 (52-100)	89 (52-100)	>10.0	3/14 (21.4)	0.667	67 (9-99)	81 (48-98)
Brainstem	Good outcome	<8.0	2/22 (9.1)	0.750	100 (16-100)	60 (36-81)	<5.0	6/13 (46.2)	1.000	100 (54-100)	100 (59-100)
	Poor outcome	>11.5	13/22 (59.1)	0.940	77 (46-95)	100 (66-100)	>5.0	7/13 (53.8)	1.000	100 (59-100)	100 (54-100)
	Mortality	>11.5	13/22 (59.1)	0.940	77 (46-95)	100 (66-100)	>5.0	6/13 (46.2)	0.952	100 (54-100)	86 (42-100)

Abbreviations: AUC=Area Under the Curve, CI=Confidence interval, CT=Computer Tomography, LSW=Last seen well

Table S5: Sensitivity and Specificity based on the ICH Volume Cut-offs from the Primary Analysis after Including Participants Who Underwent Surgical Treatment

Location	Outcome of Interest	Cut-off volume, mL	Sensitivity (95% CI)	Specificity (95% CI)
Lobar	Good outcome	<40.5	86 (71-95)	72 (62-80)
	Poor outcome	>48.0	88 (77-95)	76 (65-85)
	Mortality	>89.5	78 (63-88)	90 (82-95)
Putamen/ External Capsule	Good outcome	<32.5	88 (79-94)	71 (62-78)
	Poor outcome	>41.0	76 (65-85)	85 (77-90)
	Mortality	>42.0	89 (73-97)	75 (68-81)
Internal Capsule/ Globus Pallidus	Good outcome	<5.5	81 (65-91)	83 (70-92)
	Poor outcome	>6.0	97 (84-100)	69 (56-80)
	Mortality	>21.0	85 (62-97)	85 (75-92)
Thalamus	Good outcome	<6.5	74 (52-90)	81 (69-90)
	Poor outcome	>9.5	78 (62-90)	73 (57-85)
	Mortality	>10.5	75 (48-93)	62 (49-73)
Cerebellum	Good outcome	<17.0	58 (28-85)	59 (42-75)
	Poor outcome	>22.0	75 (53-90)	76 (55-91)
	Mortality	>22.0	89 (65-99)	74 (55-88)
Brainstem	Good outcome	<3.0	88 (47-100)	89 (71-98)
	Poor outcome	>7.5	90 (68-99)	87 (60-98)
	Mortality	>10.5	74 (49-91)	100 (79-100)

Table S6: Multivariate Analysis of Location-specific Hematoma Volume Cut-off and Neuological Outcome after Including Participants Who Underwent Surgical Treatment

Location	Outcome of Interest	Cut-off Volume, mL	ICH Volume Smaller or Larger than Cut-off	
			**Adjusted odds ratio (95% CI)**	**p**
Lobar	Good outcome*	< 40.5	12.15 (4.12 – 35.82)	**<0.001**
	Poor outcome†	> 48.0	12.24 (3.76 – 39.88)	**<0.001**
	Mortality†	> 89.5	14.37 (4.03 – 51.32)	**<0.001**
Putamen/ External Capsule	Good outcome‡	< 32.5	18.85 (6.75 – 52.65)	**<0.001**
	Poor outcome§	> 41.0	25.67 (8.32 – 79.25)	**<0.001**
	Mortality‖	> 42.0	11.95 (2.88 – 49.53)	**<0.001**
Internal Capsule/ Corona Radiata	Good outcome*	< 5.5	48.43 (10.07 – 232.89)	**<0.001**
	Poor outcome*	> 6.0	114.38 (12.37 – 1057.84)	**<0.001**
	Mortality*	> 21.0	88.09 (11.87 – 653.69)	**<0.001**
Thalamus	Good outcome#	< 6.5	15.36 (3.95 – 59.78)	**<0.001**
	Poor outcome†	> 9.5	10.89 (2.93 – 40.49)	**<0.001**
	Mortality†	> 10.5	2.53 (0.56 – 11.37)	0.227
Cerebellum	Good outcome*	< 17.0	2.76 (0.62 – 12.24)	0.182
	Poor outcome**	> 22.0	6.93 (1.74 – 27.62)	**0.006**
	Mortality¶	> 22.0	15.26 (2.51 – 92.76)	**0.003**
Brainstem	Good outcome*	< 3.0	272.57 (0.64 – 116506.90)	0.070
	Poor outcome##	> 7.5	31.04 (2.46 – 392.26)	**0.008**
	Mortality††	> 10.5	-	-

* Model was adjusted for age

† Model was adjusted for age and Glasgow Coma Scale

‡ Model was adjusted for age, Graeb score and neurosurgical treatment

§ Model was adjusted for age, Graeb score, Glasgow Coma Scale and ischemic heart disease

‖ Model was adjusted for age, Graeb score, Glasgow Coma Scale and neurosurgical treatment

Model was adjusted for age and sex

** Model was adjusted for prior antiplatelet use

¶ Model was adjusted for Glasgow Coma Scale

Unadjusted

†† Regression analysis cannot be performed as all brainstem ICH patients with volume > 10.5ml died at six months.
